# Unusual Cause of Recurrent Cholangitis: Gossypiboma

**DOI:** 10.7759/cureus.7774

**Published:** 2020-04-22

**Authors:** Bipadabhanjan Mallick, Preetam Nath, Dibya L Praharaj, Sarat C Panigrahi, Anil Anand

**Affiliations:** 1 Gastroenterology, Kalinga Institute of Medical Sciences, Bhubaneswar, IND

**Keywords:** recurrent cholangitis, gossypiboma, endoscopic retrograde cholangiopancreatography

## Abstract

Gossypiboma refers to a retained surgical sponge that can occur after any type of surgery. Though it is a rare complication of surgery, the retention of surgical sponges still occurs. We report a case of a 70-year-old woman who presented with recurrent upper abdominal pain and fever. She had a prior history of cholecystectomy and choledochoduodenostomy. Magnetic resonance cholangiopancreatography showed a filling defect in common bile duct. However, during endoscopic retrograde cholangiopancreatography and common bile duct clearance, clumps of woven fibres were removed suggestive of gossypiboma.

## Introduction

A gossypiboma refers to a surgical sponge left inadvertently in the human body following a surgical procedure [1]. It is a rare event, with reported incidences of 1 in 1,000 to 1,500 in intra-abdominal open surgery [2]. The clinical presentation is variable depending on the site of retention and type of tissue reaction, which makes the clinical diagnosis often difficult [3]. Radiograph and computed tomography can readily detect gossypiboma because of the radiopaque filaments in the surgical sponges [4]. However, this can be overlooked owing to lack of clinical suspicions and familiarity with the imaging features. We report a case of gossypiboma presenting with recurrent cholangitis.

## Case presentation

**Figure 1:**
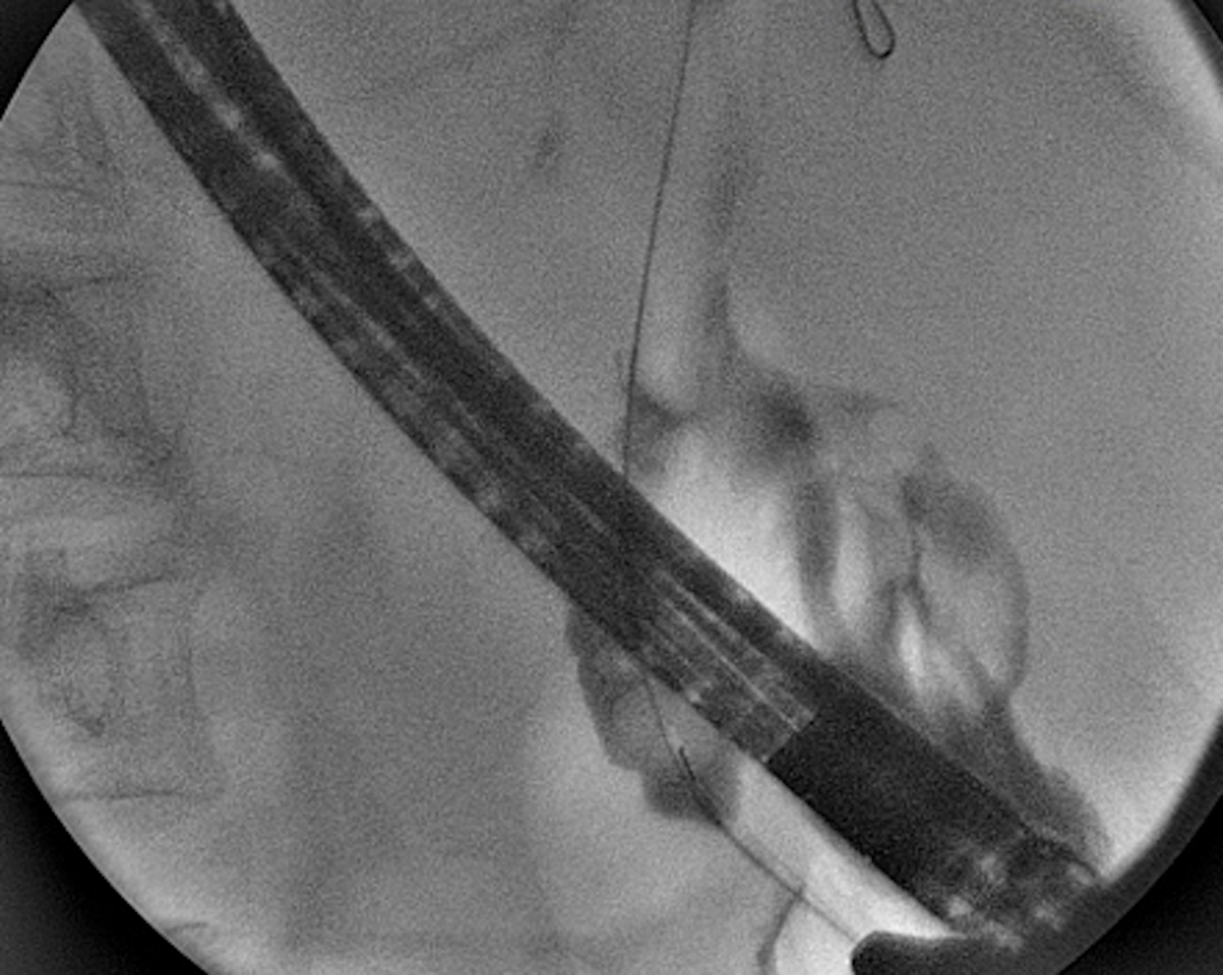
Contrast cholangiogram showing filling defect in lower common bile duct and patent choledochoduodenostomy site

A 70-year-old woman presented with upper abdominal pain and fever of one-week duration. She had similar presentation two months back to an outside hospital which was managed with antibiotics. She underwent open cholecystectomy elsewhere 20 years prior for symptomatic gallstones and choledochoduodenostomy 10 years prior for common bile duct (CBD) stones. Routine blood investigations showed raised total leukocyte count (16.5 x 10^9^/L), total bilirubin of 1.2 mg/dL, serum glutamic oxaloacetic transaminase of 23 IU/L, serum glutamic pyruvic transaminase of 37 IU/L, alkaline phosphatase of 240 IU/L (normal <104 IU/L) and gamma-glutamyl transferase of 110 IU/L (normal <39 IU/L). Contrast-enhanced magnetic resonance imaging of the abdomen with magnetic resonance cholangiopancreatography showed the presence of pneumobilia with patent choledochoduodenostomy site and filling defect in lower CBD. She was posted for endoscopic retrograde cholangiopancreatography (ERCP) and clearance of CBD. Contrast cholangiogram showed a patent choledochoduodenostomy site with filling defect in lower CBD (Figure 1).

**Figure 2:**
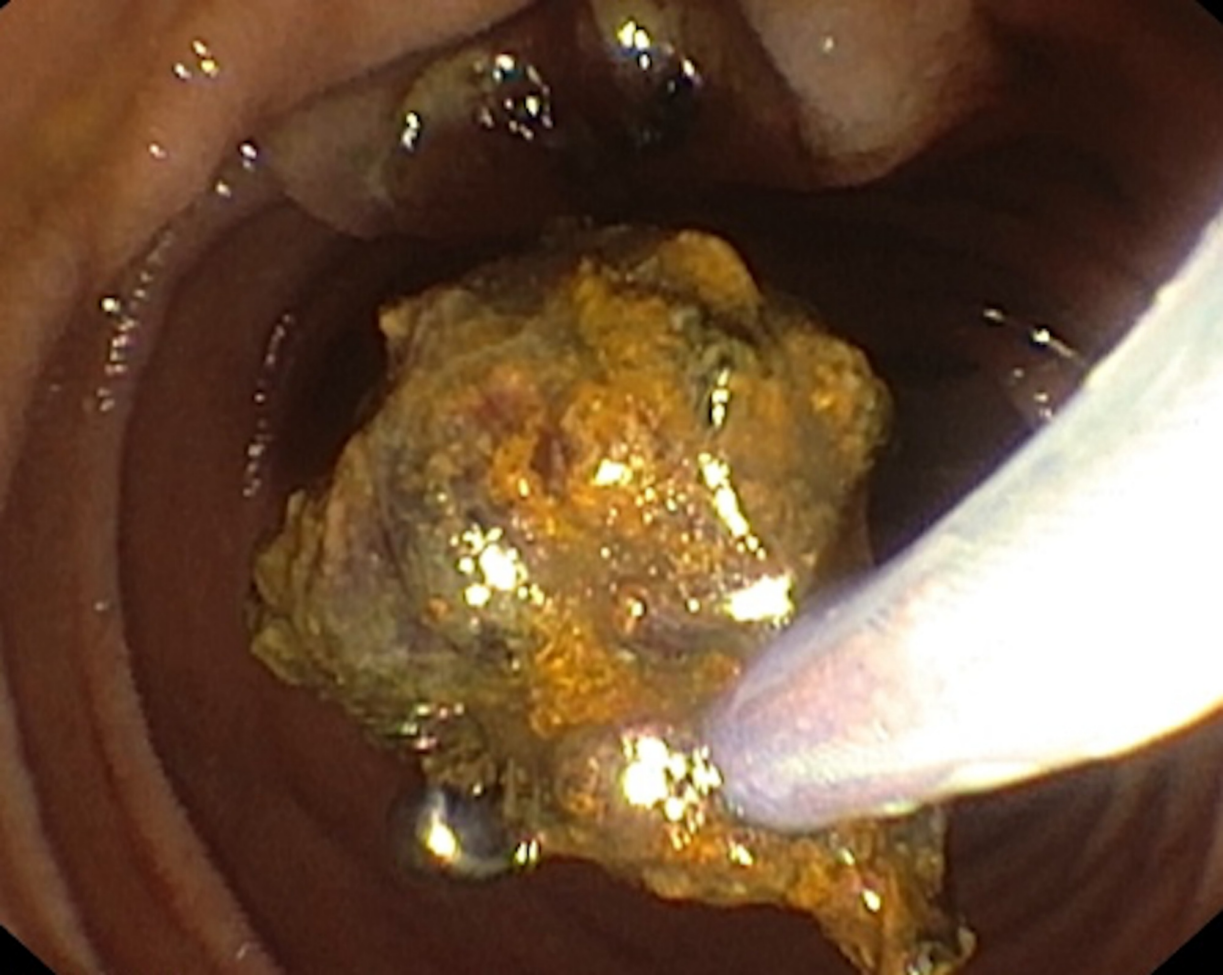
Clumps of woven fibres grasped with basket

Dormia basket was used to clear the CBD, but surprisingly clumps of woven fibres were removed (Figure 2).

**Figure 3:**
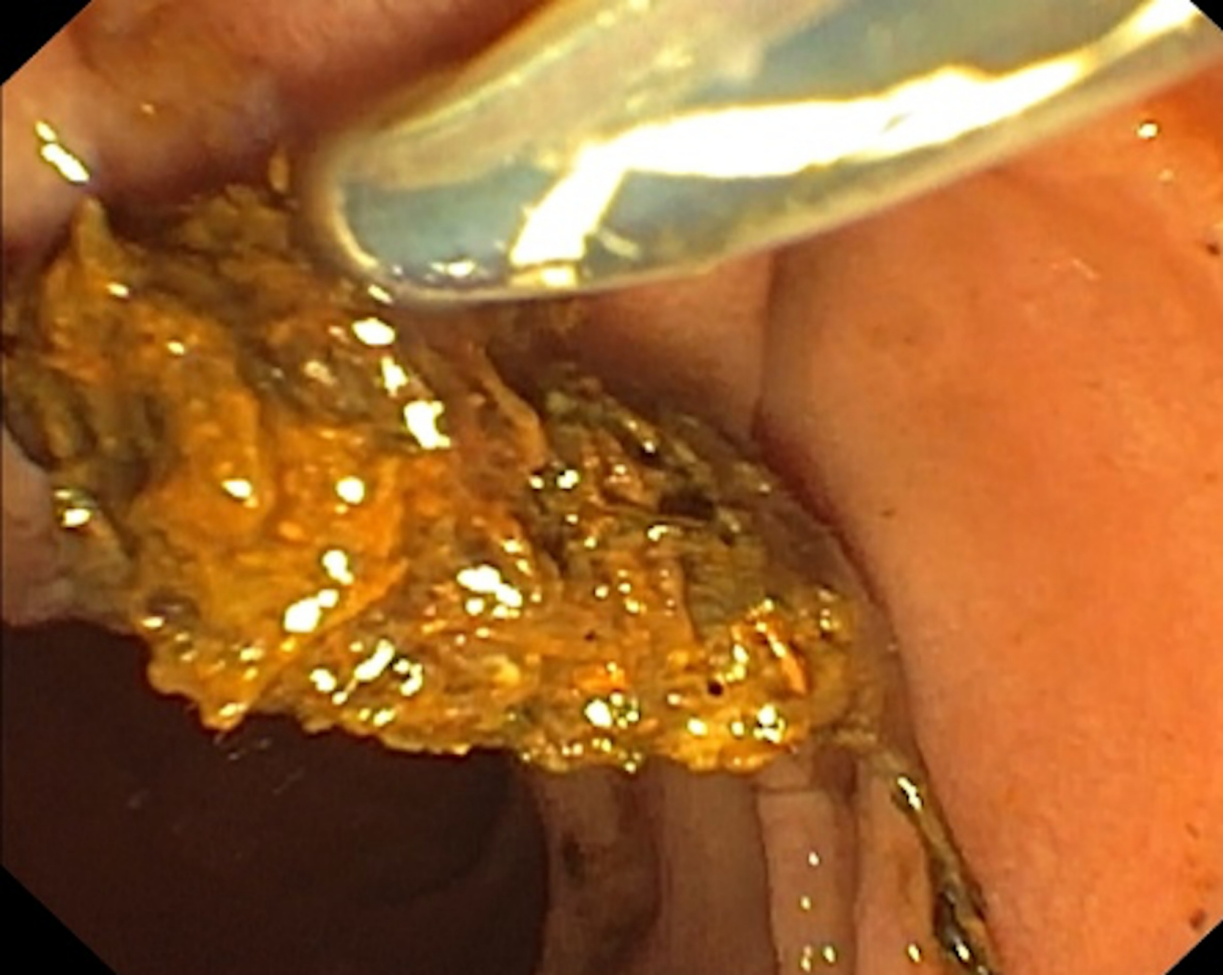
Clumps of woven fibres coming out with balloon sweep

The basket was changed to extraction balloon, and residual woven fibres were removed (Figure 3).

**Figure 4:**
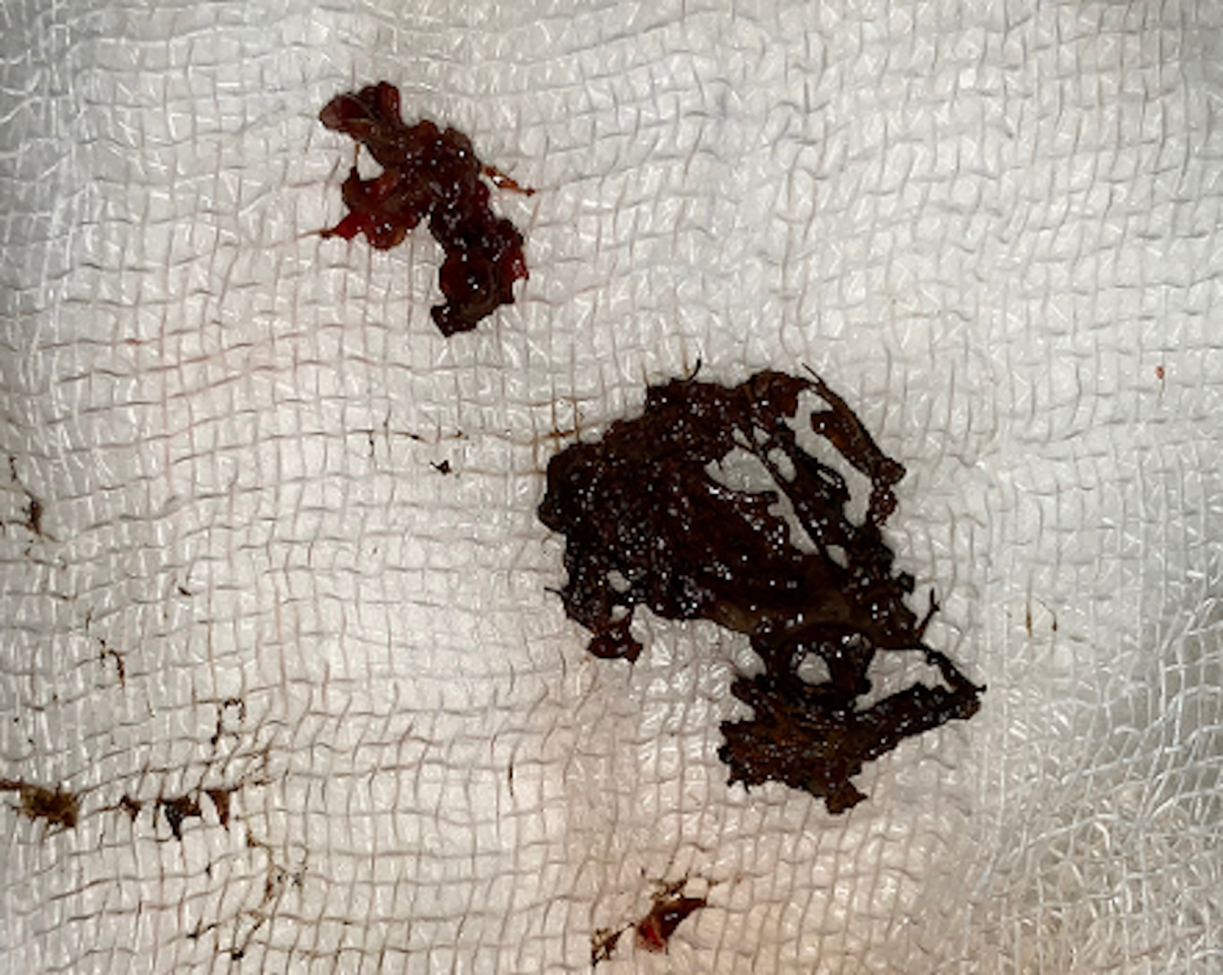
Part of the removed surgical sponge

Outside naked eye examination confirmed the retained surgical sponge (Figure 4).

At three-month follow-up, she is doing fine without any abdominal pain or fever. 

## Discussion

Gossypiboma refers to a surgical sponge left inadvertently in the human body following a surgical procedure and the subsequent tissue reaction around it [1]. Majority of gossypiboma are found in the abdomen and their clinical presentations are variable depending on the location and the type of tissue reaction [3]. The sponges are inert in human tissue and do not undergo decomposition. The retained surgical sponge causes a foreign body reaction in the form of an aseptic fibrinous response leading to formation of adhesions and encapsulation [5]. In case of high clinical suspicion, the diagnosis of gossypiboma is usually made by imaging studies [4]. The gossypiboma should be removed and surgery is the main stay of therapy [6]. Our case is unique in the sense that the gossypiboma was confined into CBD, causing recurrent cholangitis and it was removed completely by ERCP.

## Conclusions

The diagnosis of a gossypiboma is not often easy and is often clinically unsuspected and may be first recognized on imaging. Awareness of the typical radiologic appearances is critical to the diagnosis gossypiboma.
